# ﻿A remarkable new species of the flat bug genus *Nesoproxius* (Hemiptera, Aradidae), the first Oceanian representative with brachyptery

**DOI:** 10.3897/zookeys.1146.96029

**Published:** 2023-02-07

**Authors:** Shusuke Shimamoto, Seidai Nagashima, Hiroshi Nagano, Tadashi Ishikawa

**Affiliations:** 1 Laboratory of Entomology, Faculty of Agriculture, Tokyo University of Agriculture, 1737 Funako, Atsugi-shi, Kanagawa, 243-0034, Japan Tokyo University of Agriculture Atsugi-shi Japan; 2 Itami City Museum of Insects, 3-1 Koyaike, Itami-shi, Hyogo, 664-0015, Japan Itami City Museum of Insects Itami-shi Japan; 3 Japan Wildlife Research Center, 3-3-7, Kotobashi, Sumida-ku, 130-8606, Tokyo, Japan Japan Wildlife Research Center Tokyo Japan

**Keywords:** Carventinae, nymph, oceanic island, taxonomy, the Ogasawara (Bonin) Islands

## Abstract

A new flat bug species, *Nesoproxiuskishimotoi***sp. nov.**, from the Oceanian region (Ogasawara Islands, Japan) is described. It is the first brachypterous representative in the genus *Nesoproxius*. The sexual dimorphism, nymph, and habitat are also described for the first time in this genus. A key to the species of *Nesoproxius* is also provided.

## ﻿Introduction

The Ogasawara (Bonin) Islands, in the northernmost part of Micronesia, are among the Pacific islands that make up the Oceanian biogeographic region. These subtropical oceanic islands belonging to Japan are registered as a UNESCO World Natural Heritage site because of abundant endemic species with unique evolutionary patterns (UNESCO World Heritage Centre 1992; Government of Japan 2010a). The insect fauna of the Ogasawara Islands is also characterized by a high number of endemic species, accounting for approximately a quarter of the total species of insects on the islands (Government of Japan 2010a). However, many of these endemic species are endangered (Ministry of the Environment 2015) because of the various threats they face, such as damage caused by the invasive green anole (*Anoliscarolinensis* Voight, 1832) and adverse climatic conditions, including a drying trend and severe drought (Yoshida and Iijima 2009; Karube 2014; Karube et al. 2019).

Many undetermined species have recently been discovered, with some of them having been described as new endemic species (Ishikawa 2009; Ishikawa and Karube 2020; Souma and Kamitani 2020; Polhemus and Yasunaga 2021; Souma 2022), implying that the insect fauna of the Ogasawara Islands is insufficiently known. Two undetermined flat bug (Aradidae) species have been found to date (Government of Japan 2010b), and one of them, *Carventus* sp., has only been found so far in Chichijima Island. However, we have confirmed that the undetermined species belongs to the genus *Nesoproxius*, and not *Carventus* (unpublished).

*Nesoproxius* Usinger & Matsuda, 1959, a genus within the flat bug subfamily Carventinae, was originally established as a subgenus of *Proxius* Stål, 1873 and then upgraded to its current rank by Kormilev and Froeschner (1987). At present, nine species have been described from the Philippines to New Guinea, and an unidentified species has been recorded from the Ryukyu Islands, Japan (Kormilev 1983; Kormilev and Froeschner 1987; Nagashima and Shono 2012; Ishikawa 2016), thereby indicating that all known species are distributed in the Oriental and Australian regions. Additionally, all species in the genus exhibit macropterous features, which are rare among the genera in the subfamily Carventinae, as most of its members show apterous characteristics. However, all specimens from the undetermined species in the Ogasawara Islands showed brachypterous features, which helped us arrive to the conclusion that it is an undescribed species with brachypterous wings. Therefore, we describe *Nesoproxiuskishimotoi* sp. nov. as the first brachypterous and Oceanian species belonging to *Nesoproxius*. We also provide a description of the nymphs and information on the habitat of this new species, as well as an identification key to species to facilitate identification.

## ﻿Materials and methods

Most of the data were obtained from field surveys conducted by the first author in three of the Ogasawara Islands (Chichijima, Anijima, and Ototojima islands) during 2021 and 2022. These surveys which were part of a biodiversity monitoring program in a series of green anole control projects executed by the Ministry of the Environment in Japan. The remaining analyzed specimens were provided by our collaborators. Dried specimens were used for morphological observations, which were performed using a stereoscopic microscope (Olympus SZX7 and Leica M165C). All measurements were performed using a micrometer eyepiece and provided in millimeters. Illustrations were made using a stereoscopic microscope (Leica M165C) with the aid of a drawing tube (Figs 5, 6, 8).

**Figure 1.**
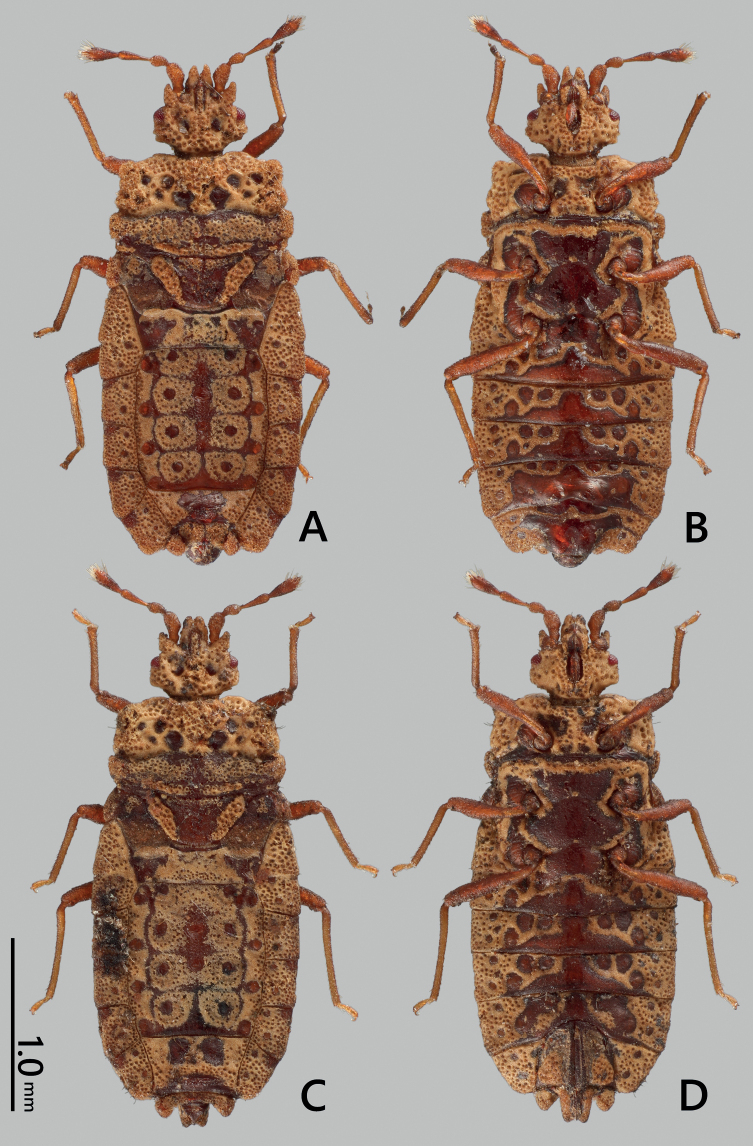
*Nesoproxiuskishimotoi* sp. nov. **A, B** male holotype **C, D** female paratype **A, C** dorsal view **B, D** ventral view.

Photographs of the specimens were taken using a digital camera (Canon EOS 5D Mark IV) with a Canon MP-E 65 mm f/2.8 1–5× macro lens (Figs 1, 3, 7), and photographs of habitats and living individuals were taken using either of two cameras: a Canon EOS 90D with a Laowa 100 mm F2.8 2× Ultra Macro APO lens or an Olympus EM-1 Mark II with a M. Zuiko digital ED 12–200 mm F3.5–6.3 lens (Figs 4, 9). Photographs of the specimens were focus-stacked using Helicon Focus 7 (Helicon Soft Ltd), and all illustrations, photographs, and images were edited using Adobe Photoshop CC (Adobe Inc.). The distribution map was created and modified by the authors with the aid of SimpleMappr (Shorthouse 2010) (Fig. 10A) and using GSI maps (Fig. 10B). Finally, the terminology used here follows that of Usinger and Matsuda (1959) and Kormilev (1968), and scientific names of plants are based on Yonekura and Kajita (2003). The specimens studied here were deposited at the Laboratory of Entomology, Tokyo University of Agriculture, Atsugi, Japan (**TUA**) and Kanagawa Prefectural Museum of Natural History, Odawara, Japan (**KPMNH**).

## ﻿Taxonomy

### ﻿Genus Nesoproxius

Taxon classificationAnimaliaHemipteraAradidae

Usinger & Matsuda, 1959

7320FFBE-EC9C-5572-B176-64E1BE1CD4B9

Nesoproxius Usinger & Matsuda, 1959: 113 (as subgenus of Proxius); upgraded to the generic rank by Kormilev and Froeschner (1987). Type species by original designation: Proxius (Nesoproxius) minutus Usinger & Matsuda, 1959.

#### Remarks.

*Nesoproxius* was previously diagnosed as a macropterous genus (Usinger and Matsuda 1959; Kormilev 1968, 1970, 1978). A brachypterous morph was found in this genus for the first time in the new species described below.

### ﻿ Nesoproxius kishimotoi

Taxon classificationAnimaliaHemipteraAradidae

Shimamoto & Nagashima sp. nov.

05551FA1-FB80-5F5D-AB4E-7D25524B8A56

https://zoobank.org/D2E26489-AF11-4034-B75E-F8CC78CBFAB7

Figs 1 , 2 , 3 , 4 , 5 , 6 , 7 , 8 , 9 Japanese name: Ogasawara-shiro-hiratakamemushi

Carventus sp.–Government of Japan 2010b: 208.

#### Type series.

***Holotype***: ♂, “Japan, Ogasawara Islands, Ototojima Island, southwest of Ainosawa, 27.1587°N, 142.1894°E, alt. ca 160 m, 11.VII.2021, Shusuke Shimamoto” (TUA).

***Paratypes*** (5 ♂ 12 ♀): **Japan, Ogasawara Islands: Chichijima Island**: 1 ♀, Renju-dani, 7.III.1999, Toshio Kishimoto (TUA); 2 ♂ 3 ♀, Renju-dani, 3.III.2022, Shusuke Shimamoto (KPMNH); 1 ♂, Nishi-kaigan, 20.VI.1999, Toshio Kishimoto (TUA). **Ototojima Island**: 2♀, same data as holotype (TUA); 1 ♂ 3 ♀, southwest of Ainosawa, 27.1591°N, 142.1899°E, alt. ca 160 m, 17.VII.2021, Shusuke Shimamoto (TUA); 1 ♂ 3 ♀, southwest of Ainosawa, 27.1591°N, 142.1899°E, alt. ca 160 m, 18.VII.2021, Shusuke Shimamoto (TUA).

#### Additional specimens examined.

Nymphs (2 spec.): **Japan, Ogasawara Islands: Ototojima Island**: 1 spec. (fourth instar), same data as holotype (TUA); 1 spec. (fifth instar), southwest of Ainosawa, 27.1591°N, 142.1899°E, alt. ca 160 m, 18.VII.2021, Shusuke Shimamoto (TUA).

#### Diagnosis.

This new species is the only brachypterous species in this genus, and it can be distinguished from all other *Nesoproxius* species by a combination of the following characters: body length approximately 3.0–3.5 mm; incrustation of body surface ocher; head vertex only slightly longitudinally raised; pronotum with only a slightly convex median ridge; scutellum trapezoidal without a median ridge; and abdomen with a relatively smooth margin.

#### Description.

**Male (holotype)** (Figs 1A, B, 5A, B). Body reddish brown, mostly covered with punctured ocher incrustations; brachypterous. Head slightly shorter than width across eyes; genae produced over tip of clypeus, slightly shorter than antennal segment I, contiguous to each other in front of clypeus; antenniferous lobes bluntly produced at apex, with parallel outer margins; postocular margins subparallel; posterolateral angles subangular, reaching level of outermost point of eye in dorsal view; vertex slightly raised longitudinally. Labium not reaching level of posterior margin of head in ventral view. Antennae 1.3 times as long as width across eyes; approximate proportion of segments I–IV 1.0: 0.7: 1.0: 1.1.

***Pronotum*** 1.9 times as wide as its length on midline, 1.3 times as long as head (excluding neck) on midline; anterior lobe strongly incrusted, with median ridge weakly inflated and slightly projected anteriad, and with four pairs of ovate smooth depressions; anterior margin slightly arched forward beyond collar at lateral one-third; anterolateral angles rounded, not projected beyond collar; lateral margins of anterior lobe convex and sinuate; posterior lobe weakly incrusted; lateral margins of posterior lobe convex anteriorly, then posteriorly concave; posterior margin weakly projected posteriorly. Scutellum trapezoidal, 0.4 times as long as its basal width, widely incrusted and elevated along lateral margins, with lateral margins straight and apex slightly rounded; median ridge thinly incrusted, slightly elevated basally; lateral incrusted fields isosceles triangular. Metanotum slightly visible behind apex of scutellum in dorsal view. Hemelytron reaching basal part of mediotergite I+II; corium reaching basal half of scutellum, projected laterally beyond lateral margin of metanotum, with posterolateral angle reflexed; hemelytral membrane rugose.

***Abdomen*** 1.4 times as long as its maximum width, with subparallel lateral margins. Mediotergite I+II mostly covered with incrustation, provided with a pair of smooth depressions laterally; mediotergites III–VI fused, weekly elevated longitudinally on midline, mostly covered with four inner pairs and three outer pairs of incrustations; inner paired incrustations each with a round smooth depression, and outer paired incrustations reaching lateral margins of respective mediotergites; mediotergite VII covered with incrustations anteriorly and laterally. Dorsal laterotergites mostly covered with incrustations, each with two round callous spots and callous outer anterolateral angle; dorsal laterotergite II+III slightly protruding at middle (posterolateral angle of original dorsal laterotergite II) and at posterolateral angle; posterolateral angles of dorsal laterotergites IV–VI not protruding; outer margin of dorsal laterotergite VI slightly angulated posteriorly; dorsal laterotergite VII posteriorly protruding and subangular, reaching level of tip of paratergite VIII in dorsal view, not reaching level of tip of pygophore. Sternite I+II covered with incrustation; sternites III–VI reticulately incrusted with small to large callosities; sternite VI with a pair of circular humps medially; sternite VII less incrusted, elevated posteromedially, with a pair of subtriangular humps medially. Paratergite VIII rhomboid, angulated posteriorly, reaching level of basal two-thirds of pygophore. Spiracles II–V ventral, spiracles VI and VII lateral, visible in dorsal view, spiracle VIII dorsolateral, visible in dorsal view.

**Figure 2.**
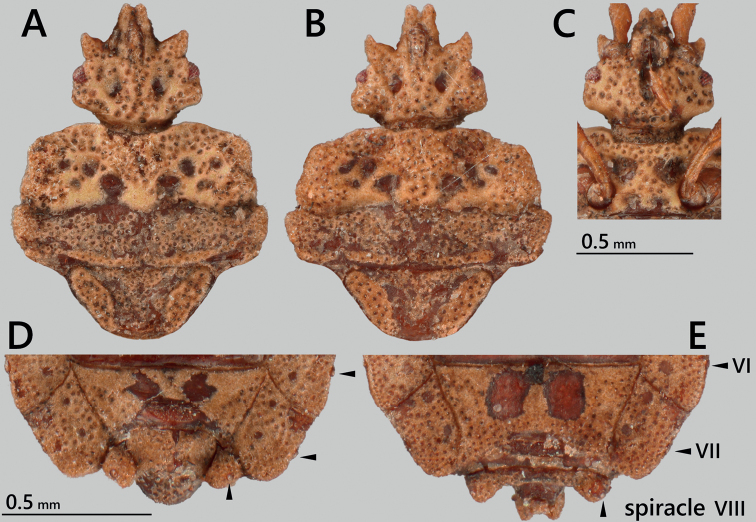
*Nesoproxiuskishimotoi* sp. nov., paratypes **A, B** head, pronotum, and scutellum, dorsal view **A** male **B** female **C** ventral view of head and pronotum **D, E** apical part of abdomen, dorsal view **D** male **E** female.

***Pygophore*** (Figs 2D, 5H) acorn-shaped, slightly shorter than its width, incrusted in basal half, scabrous in apical half.

**Female** (Figs 1C, D, 2B, E, 5B, D, G, I, K). Generally similar to male, larger than male in general; anterolateral angles of pronotum less projected; abdomen with relatively rounded lateral margins; tergite VIII subangular, nearly reaching level of basal two-thirds of paratergite IX; paratergite IX rectangular, posteriorly tricuspidate.

***Variation*** (Fig. 6). The extent of incrustations on the body surface varies among individuals as follows: posterior lobe of pronotum not incrusted (Fig. 6C, G) to completely incrusted (Fig. 6D); median part of scutellum not incrusted (Fig. 6C, F, G) to mostly incrusted (Fig. 6D); incrustations of mediotergites I+II and III–VI reduced (Fig. 6D, E) to highly developed (Fig. 6A–C, F–H); glabrous callosities of mediotergite VII commonly fused into one large smooth area (Fig. 6B, C) or rarely separated (Fig. 6A) in male, and commonly separated (Fig. 6D, E, G, H) or rarely fused (Fig. 6F) in female.

**Figure 3.**
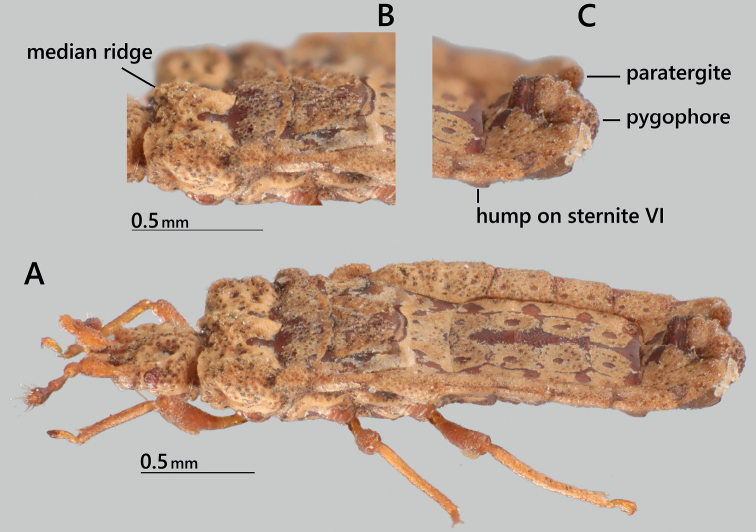
*Nesoproxiuskishimotoi* sp. nov., male paratype, dorsolateral view **A** habitus **B** pronotumand scutellum **C** apical part of abdomen.

**Figure 4.**
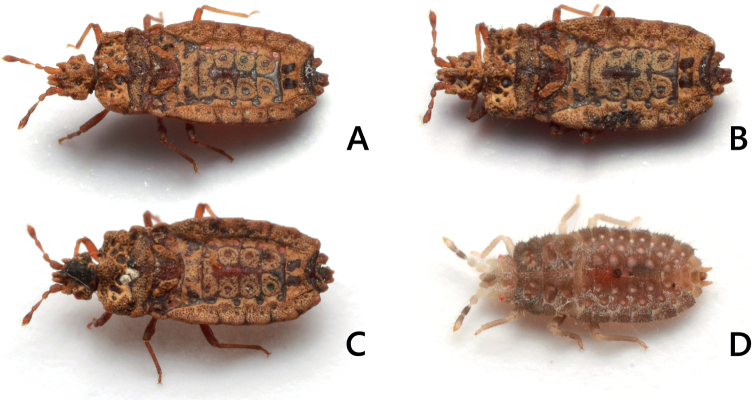
Living individuals of *Nesoproxiuskishimotoi* sp. nov. **A, B** adult female **C** same, feigning death **D** fourth instar nymph.

**Figure 5.**
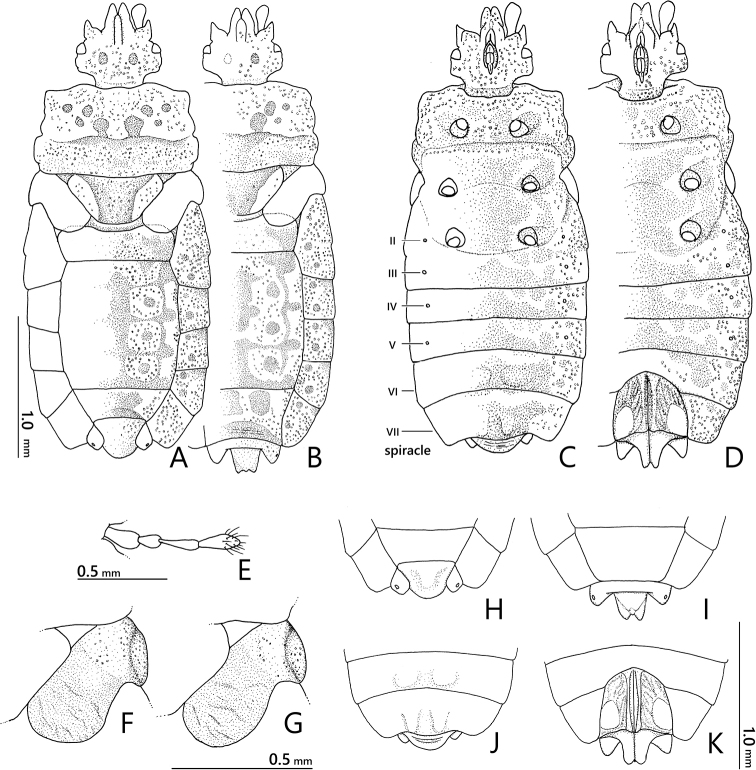
*Nesoproxiuskishimotoi* sp. nov., paratypes **A, B** habitus, dorsal view **A** male **B** female **C, D** habitus, ventral view **C** male **D** female **E** left antenna, male **F, G** hemelytra, dorsal view **F** male **G** female **H, I** apical part of abdomen, dorsal view **H** male **I** female **J, K** apical part of abdomen, ventral view **J** male **K** female.

**Figure 6.**
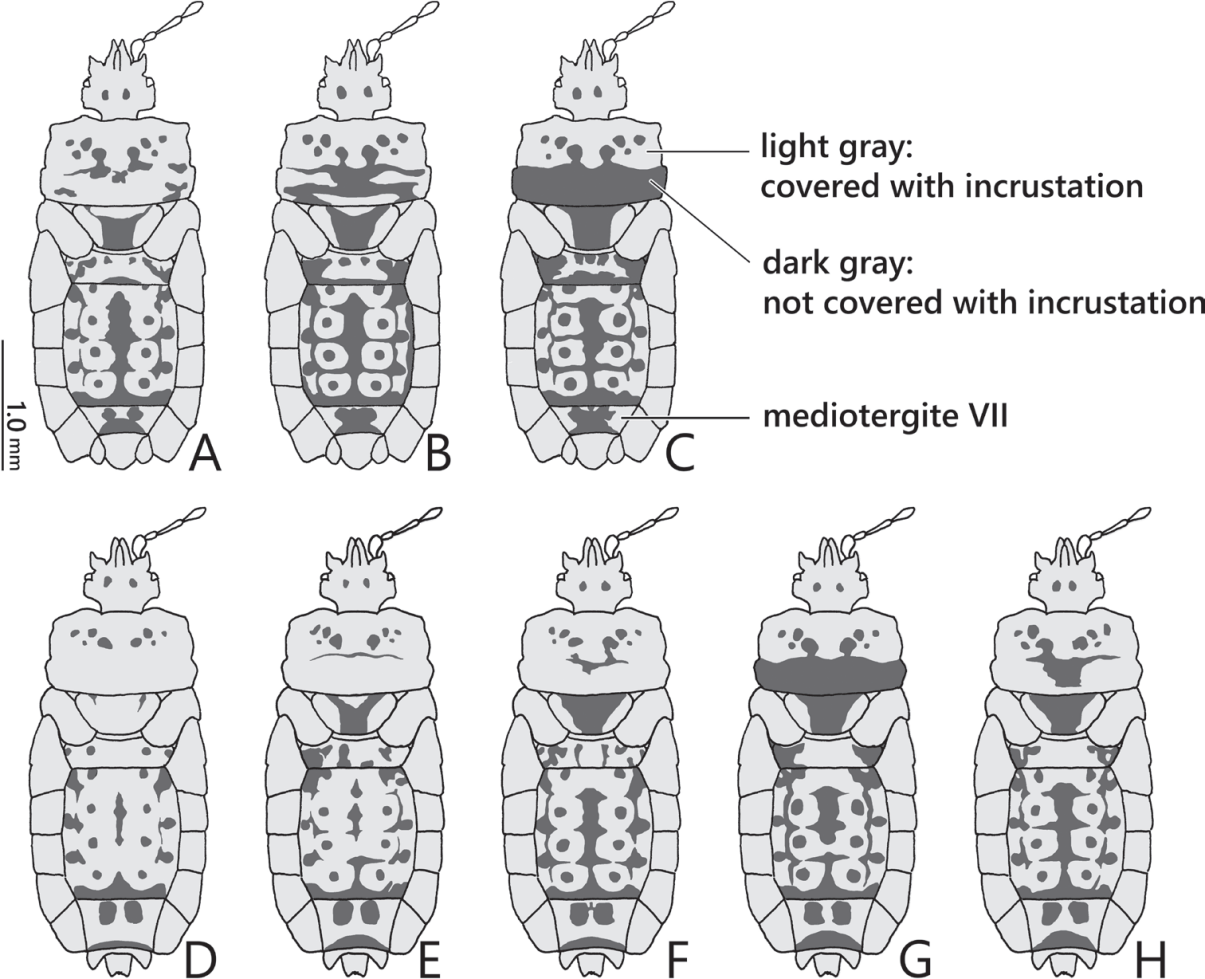
*Nesoproxiuskishimotoi* sp. nov., paratypes, variation of incrustation on head, pronotum, scutellum, and mediotergites, dorsal view **A–C** male **D–H** female.

***Measurements*** [in mm, ♂ (holotype and paratypes; *n* = 5), holotype in parentheses / ♀ (paratypes; *n* = 5)]. Body length 2.85–3.06 (2.88) / 3.06–3.47; head length 0.48 (0.48) / 0.48–0.57, width across eyes 0.55–0.57 (0.57) / 0.50–0.61; length of antennae 0.69–0.72 (0.72) / 0.70–0.80; pronotum length 0.61–0.64 (0.64) / 0.61–0.70, width 1.07–1.16 (1.11) / 1.11–1.20; scutellum length 0.32–0.36 (0.32) / 0.30–0.55, width 0.61–0.80 (0.80) / 0.68–0.93; abdomen length 1.55–1.64 (1.64) / 1.55–1.84, width 1.18–1.30 (1.27) / 1.30–1.41; pygophore length 0.23–0.25 (0.23), width 0.32–0.34 (0.32).

**Figure 7.**
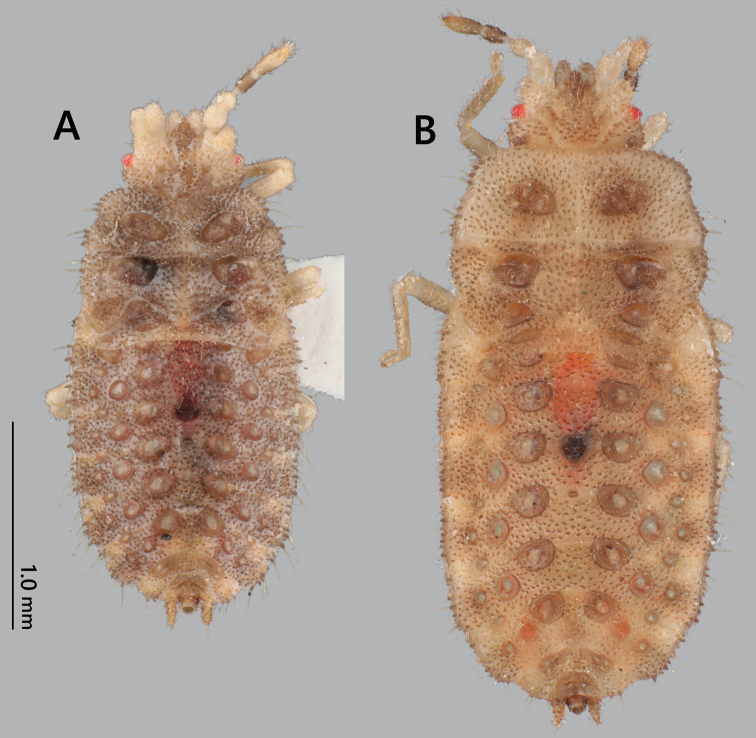
Nymphs of *Nesoproxiuskishimotoi* sp. nov. **A** fourth instar, dorsal view **B** fifth instar, dorsal view.

**Figure 8.**
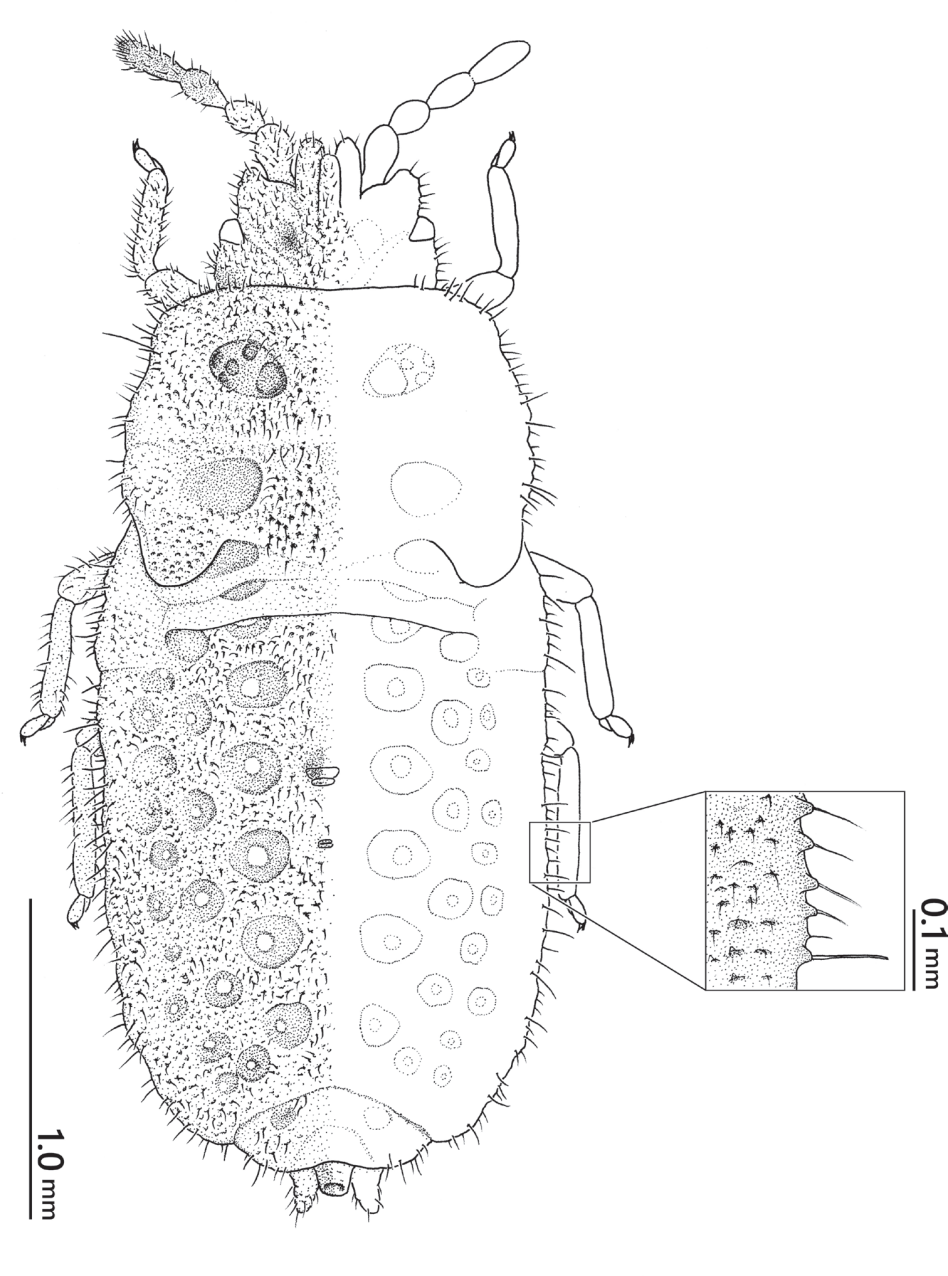
Fifth instar nymph of *Nesoproxiuskishimotoi* sp. nov., dorsal view.

***Nymph*** (Figs 7, 8). ***Fifth instar*.** Body generally beige; clypeus, vertex and posterolateral angles of head, lateral margin of thorax and abdominal segments, and center of tergites IX and X greyish beige; body length 3.3 mm; dorsum with continuously granules bearing a pubescence on apex; margin of body with larger granules bearing a longer and more erect seta on apex; head 0.6 times as long as its width on midline; antennal segment IV longest; pronotum provided with a pair of depressions, each depression with five small pits; mesonotum with a pair of smooth depressions, wing pad rounded at apex, reaching basal half of metanotum; metanotum with a pair of smooth depressions; abdominal tergites II–VI mostly not segmented; tergites I–VIII each with 1–4 pairs of round or ring-shaped depressions; two dorsal scent gland openings prominent on midline of tergum, anterior opening conspicuous and located on segment IV, posterior opening more reduced than anterior opening and located on segment V; segment IX with a pair of posteriorly elongated processes; segment X tube-shaped.

***Fourth instar*.** Generally similar to fifth instar but body generally dark gray, both sides of head beige; body length smaller, 2.6 mm; setae arising from margin of body relatively longer than fifth instar.

#### Remarks.

This new species is the first one to exhibit a brachypterous condition in *Nesoproxius*; all specimens examined showed brachypterous features, and none exhibited an apterous or macropterous condition. Even excluding the characteristics of brachypterous wings, this new species can be easily distinguished from other *Nesoproxius* species by the relatively low development of the median ridges on the pronotum and scutellum, as well as the relatively smooth abdominal margin. The unique characteristics of this new species may have been acquired through the long-term isolation in the Ogasawara Islands, which are far from New Guinea, the center of the geographic distribution of the genus.

In this study, we also clarified for the first time that sexual dimorphism in this *Nesoproxius* species is manifested in the pattern of incrustations, particularly those on mediotergite VII. Previous studies have described and illustrated this characteristic; however, all known species have been described based on one or two individuals, most of which were females (Usinger and Matsuda 1959; Kormilev 1968, 1970, 1978); therefore, identifying and describing species of this genus are necessary considering the existence of incrustations that might be the indicators of sexual dimorphism.

Moreover, this is the first time that nymphal stages have been described for *Nesoproxius* species. The body of the nymph is covered with sparse pubescence on the dorsal surface; however, it does not show the incrustations found in adults. In addition, as setae on the body margin are longer in 4^th^ instar than in 5^th^ instar nymphs, they possibly are relatively longer in younger instars.

#### Etymology.

The specific name is after Toshio Kishimoto, the first collector of this species.

#### Distribution

**(Fig. 10).** Japan: the Ogasawara Islands (Chichijima and Ototojima islands).

This new species, endemic to the Ogasawara Islands, represents the northernmost occurrence reported for *Nesoproxius*, which is far from the distribution of its congeners, and it is the first representative in this genus from the Oceanian region.

#### Habitats and biology

**Figure 9.**
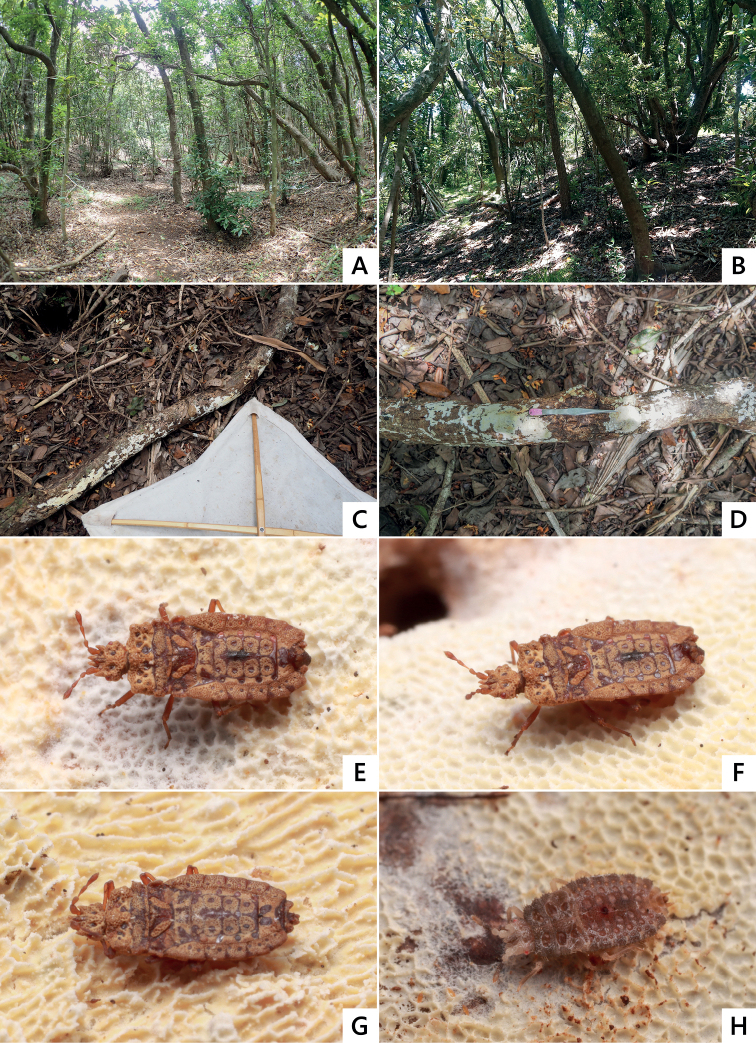
Habitats and living individuals of *Nesoproxiuskishimotoi* sp. nov. **A, B** Habitat in Ototojima Island **C, D** decayed fallen branches of *Schimawallichiimertensiana*, of which the type specimens were collected **E** adult male, dorsal view **F** ditto, dorsolateral view **G** adult female, feigning death **H** fourth instar nymph, dorsolateral view.

**(Figs 4, 9).** The new species inhabits the relatively humid forest floor of forests with tall trees, dominated by *Schimawallichiimertensiana* (Siebold & Zucc.) Bloemb. (Theaceae). However, despite our repeated field surveys, this flat bug was not found in Anijima Island, located between Chichijima and Ototojima islands (where the species inhabits), likely because, unlike the other two islands, it is entirely covered by sclerophyllous shrubs and has a dry forest floor. Therefore, it seems likely that a dry environment such as found Anijima Island is not suitable for the *N.kishimotoi* sp. nov. For this species to persists, maintaining the good condition of the ecosystems on Chichijima and Ototojima islands is necessary; however, frequent droughts in recent years may pose a challenge by negatively impacting the habitat of this species.

**Figure 10.**
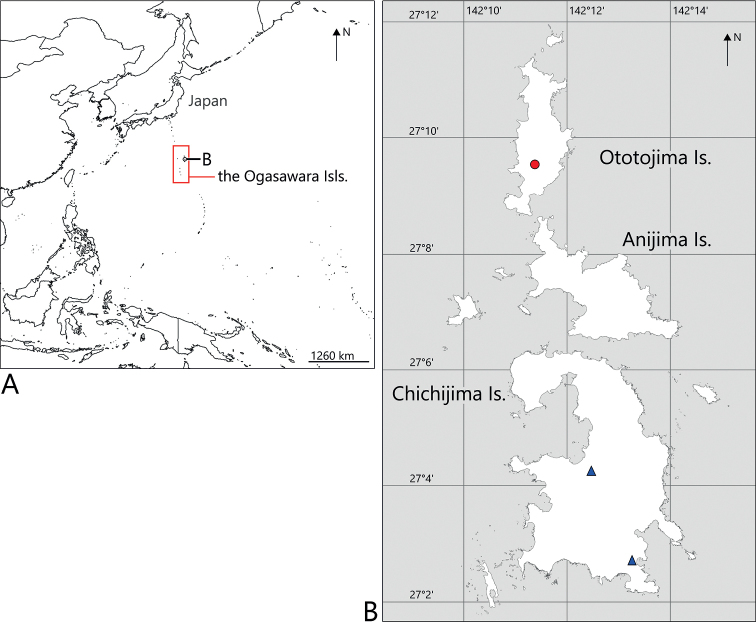
Distribution map of *Nesoproxiuskishimotoi* sp. nov. **A** location of the Ogasawara Islands **B** detail distribution in the Ogasawara Island, red circle = holotype locality; blue triangles = paratype localities.

*Nesoproxiuskishimotoi* sp. nov. was collected from the undersurface of decayed fallen branches of *Schimawallichiimertensiana* on the forest floor. Both adults and nymphs moved very slowly and frequently feigned death with folded legs and antennae. As the adults and nymphs were found together on the same branches, they all seem to inhabit the same cluster; however, their habitat range seems to be limited and scattered. The reason for this is not clear; however, it is possible that the severe damages to the soil ecosystem caused by predation by alien nemertines in the Ogasawara islands (Shinobe et al. 2017) reduce flat bug populations. Lastly, and as mentioned previously, to conserve this evolutionarily important and unique flat bug species in the Ogasawara Islands, preventing droughts and eliminating predatory alien species are necessary.

##### ﻿Key to species of the genus *Nesoproxius* (based on Kormilev 1968, 1970, 1978, 1983)

**Table:** 

1	Small species, less than 3.5 mm	**2**
–	Larger species, over 4.0 mm	**5**
2	Median ridge of scutellum clearly elevated as a T-shape	**3**
–	Median ridge of scutellum slightly elevated basally or clearly elevated longitudinally	**4**
3	Median ridge of scutellum contiguous with lateral incrusted fields posteriorly	***N.constrictus* Kormilev, 1978**
–	Median ridge of scutellum not contiguous with lateral incrusted fields posteriorly	***N.gracilis* Kormilev, 1968**
4	Anterior margin of pronotum straight; anterior angles of pronotum not projected; scutellum triangular, with median ridge clearly elevated along midline wholly	***N.minutus* Usinger & Matsuda, 1959**
–	Anterior margin of pronotum sinuate; anterior angles of pronotum projected beyond collar; scutellum trapezoidal, with median ridge slightly elevated mediobasally	***N.kishimotoi* sp. nov.**
5	Median ridge of pronotum strongly inflated, overlapping with base of head	**6**
–	Median ridge of pronotum slightly inflated, not overlapping with base of head	**8**
6	Spiracle VIII lateral	***N.malayensis* Kormilev, 1983**
–	Spiracle VIII dorsal	**7**
7	Median ridge of vertex subtriangular; median ridge of pronotum truncate posteriorly	***N.vietnamensis* Kormilev, 1968**
–	Median ridge of vertex ovate; median ridge of pronotum angulate posteriorly	***N.yoshimotoi* Kormilev, 1970**
8	Pronotum hexagonal; posterior angle of abdominal segment VII of female not reaching tip of paratergite	***N.hexagonalis* Kormilev, 1968**
–	Pronotum subrectangular or trapezoidal; posterior angle of abdominal segment VII of female reaching or exceeding tip of paratergite	**9**
9	Pronotum subrectangular, without a projection on lateral margin; posterior angle of abdominal segment VII of female not produced into a long spine	***N.punctulatus* Kormilev, 1968**
–	Pronotum trapezoidal, with a projection on lateral margin slightly before middle; posterior angle of abdominal segment VII of female produced into a long spine	***N.angulatus* Kormilev, 1968**

## Supplementary Material

XML Treatment for Nesoproxius

XML Treatment for Nesoproxius kishimotoi
